# Les infections ostéo-articulaires chez les drépanocytaires à Lubumbashi: étude épidémiologique, étiologie et prise en charge

**DOI:** 10.11604/pamj.2021.38.77.21484

**Published:** 2021-01-22

**Authors:** Manix Ilunga Banza, Nathalie Dinganga Kapessa, Augustin Kibonge Mukakala, Christelle Ngoie Ngoie, Yannick Tietie Ben N´Dwala, Vincent De Paul Kaoma Cabala, Trésor Kibangula Kasanga, Erick Wakunga Unen

**Affiliations:** 1 Département de Chirurgie, Cliniques Universitaires de Lubumbashi, Faculté de Médecine, Université de Lubumbashi, Lubumbashi, Haut Katanga, République Démocratique du Congo,; 2 Département de Chirurgie, Cliniques Universitaires de Lubumbashi, Faculté de Médecine, Université Officielle de Bukavu, Bukavu, République Démocratique du Congo

**Keywords:** Infections, ostéoarticulaires, drépanocytose, Infections, osteoarticular, sickle cell disease

## Abstract

**Introduction:**

les complications infectieuses constituent la première cause de morbi-mortalité chez le drépanocytaire surtout avant l´âge de 5 ans. Le but de cette étude est de ressortir les aspects épidémiologiques, les étiologies et la prise en charge des infections ostéo-articulaires chez les drépanocytaires à Lubumbashi.

**Méthodes:**

il s´agissait d´une étude descriptive transversale et rétrospective réalisée au centre de recherche de drépanocytose de Lubumbashi (CRDL) sur une période de trois ans soit de juin 2014 à juin 2017. Elle a porté sur tous les patients drépanocytaires suivis au CDRL ayant développé une infection ostéo-articulaire. La récolte des données s´était réalisée sur base d´une fiche d´enquête reprenant comme paramètres d´étude l´âge du patient, l´âge de la première consultation, le sexe, le motif de consultation, les antécédents, les signes physiques, le diagnostic posé, les bilans paracliniques, le traitement.

**Résultats:**

nous avions répertorié 35 cas d´infections ostéo-articulaires sur un total de 380 patients drépanocytaires soit une fréquence 9,2%. La tranche d´âge la plus touchée était celle de moins de 5 ans (37,1%); l´âge moyen était de 10,9±9,5 ans pour des extrêmes de 8 mois et 37 ans. Il y a une légère prédominance féminine avec 51,4% des cas (sexe ratio de 1,06 en faveur des femmes). Les antécédents majeurs étaient la transfusion (16,6%) et la splénectomie (8,6%). Le motif de consultation était dominé par la douleur des membres (84%). Vingt patients (57,1%) avaient des conjonctives bulbaires ictériques et 26 (74,3%) étaient pâles. A l´inspection, la tuméfaction des membres et la plaie étaient retrouvées respectivement chez 27 patients (77,1%) et 19 patients (54,3%). La splénomégalie cliniquement palpée chez 6 patients (17,1%). Trois types d´infections ostéo-articulaires avaient été décelés, dominés par l´ostéomyélite avec 24 cas (68,57%) suivi de l´ostéite avec 7 cas (20%) et l´arthrite suppurée avec 4 cas (11,43%). Sur les 24 cas d´ostéomyélites, 18 étaient aiguës (75%) et 6 étaient chroniques (25%) dont 4 hyperostosantes et 2 fistulisantes. Le tibia a été l´os le plus touché (18 cas), la radiographie était dominée par des images d´ostéolyse avec 27 cas (77,1%) puis de réaction périostée avec 15 cas (42,9%). Le type homozygote était retrouvé chez 88,6% des cas. L´hémoculture a été réalisée chez 17 patients sur les 35 et la salmonelle a été isolée dans 15 cultures sur les 17 soit 88,23% alors que la pyoculture réalisée chez 10 patients avaient isolé également d´autres germes. Le bilan inflammatoire avait été réalisé chez 21 patients dont 15 avaient une hyperleucocytose, 13 une formule leucocytaire pathologique, tous avaient une vitesse de sédimentation accélérée (supérieure à 20mm à la 1^ère^ heure). Concernant le calendrier vaccinal, les vaccins du PEV étaient suivis par 62,86% des patients alors que le taux de vaccination spécifique pour les drépanocytaires était de 17,14%. Sur le plan thérapeutique, tous nos patients avaient reçu un traitement médical, 6 chirurgicaux (17,14%) ayant consisté en une séquestrectomie et la majorité des patients avaient été traités orthopédiquement (25 cas). **Conclusion:** l´infection osseuse chez les drépanocytaires reste une réalité très préoccupante dans notre milieu pauvre ou la plupart n´ont pas une vaccination préventive spécifique aux drépanocytaires.

## Abstract (en)

**Introduction:**

infections are the leading cause of morbidity and mortality in patients with sickle cell disease, especially before age 5 years. The purpose of this study was to highlight the epidemiological features, etiologies and management of osteoarticular infections in patients with sickle cell disease in Lubumbashi.

**Methods:**

we conducted a descriptive, cross-sectional and retrospective study at the Research Center for Sickle Cell Disease in Lubumbashi (RCSCDL) over a three-year period from June 2014 to June 2017. It included all patients with sickle cell disease on follow up at the RCSCDL who developed osteoarticular infection. Data were obtained from a survey form. Parameters were patient’s age, age at first visit, sex, reason for consultation, history, physical signs, diagnosis, paraclinical assessment and treatment.

**Results:**

we identified 35 cases of osteoarticular infections out of a total of 380 cases of sickle-cell disease, reflecting a rate of 9.2%. The most affected age group was people under 5 years of age (37.1%); the average age was 10.9±9.5 years ranging from 8 months and 37 years. There was a slight female predominance (51.4% of cases; sex ratio 1.06 in favor of women). Most patients with osteoarticular infection had a history of transfusion (16.6%) and splenectomy (8.6%). The most common reason for consulting was limb pain (84%); 20 patients (57.1%) had bulbar conjunctival icterus and 26 (74.3%) were pale. Clinical examination showed limb swelling and wound in 27 patients (77.1%) and 19 patients (54.3%), respectively. Clinical palpation of the splenomegaly was performed in 6 patients (17.1%). Three types of osteoarticular infections were detected. They were dominated by osteomyelitis (24 cases; 68.57%) followed by osteitis (7 cases; 20%) and suppurative arthritis (4 cases; 11.43%). Out of 24 cases of osteomyelitis, 18 were acute (75%) and 6 were chronic (25%), of which 4 had a hyperostosing behaviour and 2 a fistulising behaviour. Tibia was the most affected bone (18 cases), X-ray mainly showed osteolysis (27 cases; 77.1%) and then periosteolysis (15 cases; 42.9%). Homozygous sickle cell disease was found in 88.6% of cases. Hemoculture was performed in 17 out the 35 patients and salmonella was isolated in 15 out of 17 cultures (88.23%). Pyoculture was performed in 10 patients; it isolated other germs. Assessment of inflammation was performed in 21 patients: 15 had hyperleukocytosis, 13 pathological white blood cell formula , all had increased sedimentation rate (greater than 20mm in the 1st hour). With respect to immunization schedule, 62.86% of patients received EPI vaccines while patients with sickle cell disease who needed specific vaccine had an adherence rate of 17.14%. With respect to therapy, all of our patients received medical treatment; 6 patients underwent sequestrectomy (17.14%) while the majority of patients (25 cases) underwent orthopedic treatment. **Conclusion:** bone infection in patients with sickle cell disease is a worryng issue in our poor environment where there isn’t a specific vaccine for patients with sickle cell disease.

## Introduction

La drépanocytose est une maladie génétique autosomique récessive caractérisée par la présence d´une hémoglobine anormale, qui favorise la falciformation irréversible des hématies en situation d´hypoxie prolongée et aboutissant à des manifestations vaso-occlusives [1]. C´est une affection caractérisée par sa grande fréquence (plus d´un million d´individus atteints dans le monde). Piel estime sur base des données des Nations unies que 307630 bébés sont nés avec la drépanocytose en 2010 [2]. De nombreuses complications aiguës et chroniques, causes de morbidité font parties intégrantes de cette affection. Les complications aiguës sont principalement représentées par les crises vaso-occlusives, l´anémie hémolytique chronique avec des épisodes d´aggravation aiguë et la susceptibilité aux infections bactériennes à germes encapsulés en raison de l´asplénie fonctionnelle [1, 3-6] et un déficit en complet [6]. Les complications infectieuses sont une grande source de morbidité et de mortalité chez les drépanocytaires [7]. De fait, la drépanocytose est caractérisée par une extrême sensibilité aux infections par les bactéries encapsulées, spécifiquement pneumocoques et aussi *Salmonella* en Afrique. Malgré le traitement préventif anti-infectieux, l´effet n´est pas complet, et les complications infectieuses restent fréquentes chez des nombreux sujets drépanocytaires [8].

Dans une étude récente réalisée dans notre milieu sur les pathologies digestives chez les drépanocytaires de Lubumbashi, Manix *et al*. ont trouvé que les atteintes osseuses représentaient 70,87% et venaient en troisième position après celles respiratoires et oto-rhino-laryngologiques [9]; la pathologie osseuse est très fréquente chez les drépanocytaires et dominée par deux grandes entités: l´infarctus et l´infection; l´infarctus étant beaucoup plus fréquent (environ un ratio de 50/1) [10]. Van Der Have affirme que les principales manifestations orthopédiques chez les drépanocytaires sont les ostéonécroses, les ostéomyélites, les arthrites septiques et les infarctus osseux [11] alors que chez les sujets non drépanocytaires, les atteintes osseuses sont dominées par l´ostéomyélite aiguë hématogène et l´arthrite septique et les germes les plus courants sont le *Staphylocoque aureus et le Kingella kingae* [12]; chez les drépanocytaires, les ostéonécroses et les ostéomyélites sont parmi les plus sérieuses complications musculo-squelettiques et les plus importants handicap [11], et les germes les plus souvent impliqués sont par ordre de fréquence décroissante les *Salmonella*, le *Staphylocoques aureus* puis les bacilles gram négatif (*E. coli*) pour les ostéomyélites tandis que pour l´arthrite septique les germes encapsulés tels que le pneumocoque et l´*Haemophilus influenzae* sont les plus incriminés du fait de l´hyposplénisme [13]. Cependant certaines controverses existent concernant la bactériologie, l´étiologie et la présentation clinique en différenciant l´infection ostéo-articulaire de l´infarctus osseux [14].

Le traitement efficace de l´infection ostéo-articulaire chez les drépanocytaires requiert un diagnostic précoce, une compréhension de la physiopathologie de la maladie et la connaissance des alternatives de traitement médical et chirurgical [11]. L´infection osseuse étant difficile à manager, le traitement préventif s´impose. Vu la susceptibilité aux infections aux germes encapsulés des drépanocytaires, la vaccination non seulement ordinaire selon le programme élargie de vaccination (PEV) mais aussi et surtout celle spécifique aux drépanocytaires qui comprend le vaccin anti-pneumococcique (PNEUMOVAX 23), anti-salmonelle (TYPHIM), anti-méningocoque A et C et anti-haemophilus de type b.

Il existe de nombreuses littératures publiées sur la drépanocytose à travers le monde orientées pour la plupart vers les infections en général mais peu d´entre elles abordent les aspects sur les infections osseuses chez les drépanocytaires de tout âge et aucune donnée récente dans notre région n´est disponible concernant les infections ostéo-articulaires du drépanocytaire; c´est donc dans ce cadre que nous avions orienté notre étude dans ce sens avec comme objectif de disponibiliser les données épidémiologiques, les types d´infections osseuses et leurs germes dans notre milieu ainsi que leur prise en charge.

## Méthodes

### Matériels

#### Description du cadre d´étude

Notre étude s´est déroulée à l´Institut de recherche en Sciences de la Santé en sigle I.R.S.S. qui est un établissement public à caractère scientifique et technique. Par le biais du Centre de Référence de la Drépanocytose de Lubumbashi en sigle « CRDL », situé dans l´enceinte de l´Hôpital Général Provincial de Référence Jason Sendwe de Lubumbashi (salle 5) où il s´occupe des consultations, de traitement et suivi de plusieurs patients drépanocytaires de tout âge et des différentes contrées.

### Patients

#### Population d´étude

Notre étude a porté sur les patients drépanocytaires de tout âge et de tout sexe suivis au centre de référence de la drépanocytose de Lubumbashi « CRDL » pour une pathologie infectieuse ostéo-articulaire, durant notre période d´étude.

### Taille de l´échantillon

Composée d´un total de 380 patients drépanocytaires ayant consulté le CRDL de l´an 2015 à 2017 soit 3 ans dont 291 avaient présenté des complications infectieuses, parmi lesquelles 35 avaient des infections ostéo-articulaires diagnostiquées. Ils proviennent des 7 communes de la ville de Lubumbashi mais aussi de ses environs et en dehors (Likasi, Kindu, Kasumbalesa) et en dehors du pays (Ndola-Zambie) et sont retenus pour cette étude selon les critères de sélection ci-dessous:

### Les critères d´inclusion et de non inclusion

Ont été retenus: tous les patients drépanocytaires selon la clinique ou la paraclinique (Test d´Emmel et électrophorèse) suivis au CRDL pendant 3 ans (2015, 2016 et 2017); Tous les patients drépanocytaires ayant fait au moins une pathologie infectieuse ostéo-articulaire pendant cette période de suivi de 3 ans et ayant tous les paramètres étudiés par le CRDL; Il s´agit d´une **ostéomyélite aiguë** (infection par voie hématogène, localisation le plus souvent au niveau de la métaphyse fertile de l´enfant associant fièvre, douleur ostéo-articulaire, absence de suppuration chronique), une ostéomyélite chronique (soit une évolution d´une ostéomyélite aiguë ou une ostéomyélite d´emblée, associant un séquestre et/ou une fistule avec suppuration chronique, la fièvre pouvant être absente), une **ostéite** (inflammation de l´os localisée limitée à la corticale avec une cavité médullaire radiologiquement saine, le plus souvent non métaphysaire, ou chez les adultes qui n´ont plus de métaphyse fertile, acquise le plus souvent par voie locale ou de proche en proche donnant des douleurs osseuses, la fièvre manque le plus souvent, des images d´ostéolyses radiographiques), l´**arthrite suppurée** (association de fièvre, douleur, impotence fonctionnelle, tuméfaction articulaire, avec présence d´un liquide purulent intra-articulaire objectivée par une ponction diagnostique, lorsque ces manifestations sont accompagnées de modifications de l´épiphyse osseuse, cela devient une ostéo-arthrite).

N´ont pas été retenus: tous les patients n´ayant pas fait une infection ostéo-articulaire pendant les 3 ans de suivi; tous les patients ayant une autre atteinte ostéo-articulaire qui n´est pas une infection notamment une ostéonécrose aseptique (est la condition associant à différente étapes l´ostéoporose, une géode et une déformation de l´épiphyse sans infection), une **arthrite réactionnelle non suppurée du drépanocytaire, une crise vaso-occlusive osseuse**; tous les dossiers incomplets n´ont pas été retenus dans notre étude.

**Type et période d´étude:** notre étude, est une étude descriptive transversale et rétrospective allant du 1^er^ janvier 2015 au 31 décembre 2017.

**Paramètres d´étude:** nous avons étudié les paramètres suivants: 1) Variables épidémiologiques et socio-démographiques: âge de première consultation, sexe, provenance; 2) Variables cliniques: motif de consultation, antécédents, signes physiques et diagnostic posé; 3) Variables paracliniques: test d´Emmel, électrophorèse d´hémoglobine pour le diagnostic de drépanocytose et la radiographie, la biologie pour les infections ostéo-articulaires; 4) Variables thérapeutiques: traitement préventif et curatif.

### Technique de récolte des données

La collecte des données pour cette étude a été faite à l´aide d´une fiche d´enquête sur papier remplie au crayon et stylo grâce aux informations recueillies après lecture des dossiers médicaux des patients drépanocytaires conservés soigneusement au CRDL.

### Saisie des données et analyses statistiques

Les données recueillies ont été saisies grâce au logiciel Microsoft Excel et, l´analyse a été facilitée par le logiciel Epi-info 7.2 qui nous a permis de déterminer des mesures de fréquences (absolues et relatives) ainsi que des paramètres de position et de dispersion. Pour l´étude de l´association statistique, nous avons utilisé le test de chi-carré corrigé de Yates et la valeur du « p », dont le seuil de signification était fixé à 5%.

**Présentation des résultats:** les résultats seront présentés sous forme des écrits mais aussi sous forme des tableaux.

**Considérations éthiques:** nous avons observé les principes d´anonymat et de confidentialité, en ce qui concerne les identités de nos patients.

## Résultats

Au cours de notre période d´étude, nous avions répertorié 35 cas d´infections ostéo-articulaires sur un total de 380 patients drépanocytaires, ce qui représente une fréquence de 9,2%. Par rapport aux complications infectieuses, 291 patients ont développé une infection quelle qu´elle soit sur un total de 380 suivis soit 12%. En fonction des tranches d´âge, la tranche d´âge la plus touchée est celle qui va de 0 à 5 ans avec 37,1% des cas suivie par celle de 6 à 10 ans avec 22,9% des cas; l´âge moyen étant de 10,9 ± 9,5 ans, pour des extrêmes de 8 mois et 37 ans; le sexe féminin prédomine légèrement avec une fréquence de 51,4% des cas contre 48,06% des cas masculins; le sexe ratio était de 1,06 en faveur des femmes; mais la différence statistique n´est pas significative entre le sexe et la survenue des infections ostéo-articulaires (p = 1,000) (Tableau 1). Sur nos 35 patients, 5 seulement avaient un antécédent de transfusion et l´analyse des facteurs associés entre la transfusion et la survenue des infections ostéo-articulaires montre qu´il n´y a pas de corrélation statistique significative (p = 1,000). Deux signes cliniques ont constitué le motif de consultation à savoir la douleur des membres et la difficulté de marcher avec respectivement 84,0 et 39,0% des cas.

**Tableau 1:** répartition des cas en fonction de l´âge et du sexe

Variables	Effectif	Pourcentage (%)
**Tranche d´âge (ans)**		
[0-5[	13	37,1
[5-10[	8	22,9
[10-15[	3	8,6
[15-20[	5	14,3
[20-50[	3	8,6
[25-30[	1	2,9
[30-35[	1	2,9
[35-40[	1	2,9
Total	35	100
**Sexe**	**Effectif**	**Pourcentage**
Masculin	17	48,6
Féminin	18	51,4
Total	35	100

La tranche d´âge de 0-5 ans est la plus représentée; âge moyen 10,9±9,5 ans; âges extrêmes: 8 mois et 37 ans. Légère prédominance du sexe féminin avec 51,4%; sexe ratio F/H =1,06

L´anémie aiguë avait été retrouvée chez 27 patients soit 77,14% des cas alors que seulement 8 patients soit 22,86% des cas n´ont pas développé une anémie aiguë; la fièvre a été noté chez 16 patients (45,71%). A l´inspection du membre touché, la tuméfaction avait été retrouvée chez 27 patients soit 77,1% des cas alors que une plaie avait été retrouvée chez 19 patients soit 40% des cas. Selon les types d´infections ostéo-articulaires diagnostiqués, les ostéomyélites sont les plus importantes et ont représenté 24 cas soit 68,57% des cas dont 18 cas de forme aiguë et 6 chroniques (Tableau 2). En fonction des os et articulations touchés, le tibia a été le plus touché avec 18 cas (51,42%), ensuite l´humérus avec 6 cas (17,14%), les os de l´avant-bras (radius et/ou cubitus) avec 5 cas (14,28%), le fémur et les os de la main avec 1 cas chacun (2,85%). Les arthrites infectieuses ont concerné la cheville chez 3 patients (8,57%) et le poignet 1 patient (2,85%) (Tableau 3).

**Tableau 2:** répartition des cas en fonction du type d´infection osseuse

Type d´infection	Effectif	Pourcentage (%)
ostéomyélites	24	68,57
Aiguës	18	51,42
Chroniques	6	17,14
Ostéites	7	20,00
Arthrites suppurées	4	11,43
Total	35	100


Les ostéomyélites sont les infections osseuses les plus fréquentes avec 68,57%

**Tableau 3:** répartition de l´infection ostéo-articulaire en fonction de la localisation

Variables		
**Arthrite suppurée**	**Effectif**	**Pourcentage (%)**
Cheville	3	75
Poignet	1	25
**Total**	**4**	**100**
La cheville est la plus touchée par l´arthrite suppurée avec 75%		
**Ostéomyélite**	**Effectif**	**Pourcentage (%)**
Fémur	1	4,16
Tibia	11	45,83
Péroné	1	4,16
humérus	5	20,83
Tibia et péroné	2	8,32
Radius et/ou cubitus	3	12,48
Carpes de la main	1	4,16
**Total**	**24**	**100**
Le tibia est le plus touché par l´ostéomyélite avec 45,83%		
**Ostéites**	**Effectif**	**Pourcentage**
Tibia	4	57,14
humérus	1	14,28
Radius et/ou cubitus	2	28,56
**Total**	**7**	**100**

Le tibia est le plus touché par l´ostéite avec 57,14% des cas

A la radiographie, les images d´ostéolyse ont été les plus retrouvées avec 27 cas soit 77,1% des cas suivi des images de réactions périostées avec 15 cas soit 42,9% des cas et enfin les images de séquestre avec 6 cas soit 17,1%. En fonction du trait drépanocytaire, les homozygotes (SS) étaient majoritaires avec 31 cas sur 35 soit 88,6% alors que le trait hétérozygote (AS) n´a été retrouvé que chez 4 patients soit 11,4% des cas. L´association entre le trait drépanocytaire et les infections ostéo-articulaires montre que le trait drépanocytaire influence la survenue des infections ostéo-articulaires de façon statistiquement significative (p=0,000 et OR = 39,5) (Tableau 4). En fonction de la biologie réalisée, l´hémoculture a été réalisée chez 17 patients soit 48,6% des cas et la *Salmonelle* a été isolée dans 15 cultures sur les 17 et *Escherichia coli* dans les 2 autres cultures tandis que la pyoculture réalisée chez 10 patients a donné 2 cultures stériles (20%), le *Staphylocoque aureus* chez 3 patients (30%), la *Salmonelle* chez 2 patients (20%), le *Streptococcus pneumoniae* chez 2 patients( 20%), le *Klebsiella* chez 1 patient(10%); le bilan inflammatoire réalisé chez 21 patients a révélé une hyperleucocytose chez 15 patients, une formule leucocytaire pathologique chez 13 patients et une vitesse de sédimentation accélérée (au-delà de 10 mm à la 1ère heure) chez tous les 21 patients.

**Tableau 4:** répartition des patients selon le trait drépanocytaire et l´infection ostéo-articulaire

Variables	Infections ostéo-articulaires			
Trait drépanocytaire	Non	Oui	Total	P value
Homozygote	158 (83,6%)	31 (16,4%)	189 (100,0%)	0,000 OR: 39,5
Hétérozygote	98 (96,1%)	4 (3,9%)	102 (100,0%)	
Total	256 (88,0%)	35 (12,0%)	291 (100,0%)	

16,4% des patients homozygotes ont développé une infection ostéo-articulaire contre 3,9% des hétérozygotes; cette différence est statistiquement significative (p = 0,000)

Le calendrier vaccinal du Programme élargi de vaccination (PEV) avait été suivi par 62,86% des patients alors que pour les vaccins spécifiques EUVAX/ENGERIX, ACT-HIB, TYPHIM IV, seulement par 6 patients (17,14%). En dépit du traitement médical suivi par tous nos 35 patients fait des antibiotiques pendant une période d´au moins 4 semaines et des antalgiques, un traitement chirurgical avait été appliqué chez 10 patients (28,58%) ayant consisté en une séquestrectomie pour 6 malades et incision-drainage articulaire pour les 4 patients avec arthrites suppurées et les 25 patients restants (71,42%) étaient traités orthopédiquement par un matériel plâtré; et le traitement de fond à l´hydroxyurée suivi par uniquement 5 patients (14,28%). La répartition des patients en fonction de l´âge et du type d´infection ostéo-articulaires montre la prédilection de l´ostéomyélite chez les patients âgés de 6 à 10 ans avec 8 cas, l´arthrite plus dans la tranche d´âge de 21 à 25 ans avec 2 cas et l´ostéite également chez les moins de 5 ans avec 3 cas. La différence statistique est significative (p = 0,0326).

## Discussion

### Données épidémiologiques

Les infections sont fréquentes chez les drépanocytaires. La susceptibilité aux infections du drépanocytaire est connue. Elle est attribuée à l´asplénie fonctionnelle qui est la conséquence d´infarctus splénique répétés [15]. Les complications infectieuses sont une grande source de morbidité et mortalité chez le drépanocytaire [7]. Sur un total de 380 patients drépanocytaires, nous avons trouvé une pathologie infectieuse chez 291patients soit une fréquence de complications infectieuses de 6,57% des cas. Parmi ces infections, celles osseuses ont représenté 35 cas sur les 291 soit 12% de l´ensemble des infections et ce qui correspond à 9,2% de l´ensemble des drépanocytaires. Notre fréquence est de loin inférieure à celle de DIOP [7] qui, travaillant sur les infections bactériennes chez 100 drépanocytaires fébrile a trouvé une fréquence de 24% pour les os derrière l´infection oto-rhino-laryngologique et pulmonaire avec respectivement 32 et 28%; sa fréquence élevée s´explique par le fait qu´il a exclu dans ce calcul le paludisme qui représente chez lui 48% et n´a calculé que les infections bactériennes alors que notre fréquence a pris en compte également les cas de paludisme.

### Données socio-démographiques

**En rapport avec l´âge:** les enfants de moins de 5 ans ont représenté le plus grand effectif avec 13 cas sur 35 soit 37,1% et la tranche d´âge de 6 à 10 ans 8 cas soit 22,9% des cas. Les âges extrêmes de notre série sont de 10 mois et 37 ans pour une moyenne d´âge de 10,9 ± 9,5 ans. Notre moyenne d´âge est supérieure à celle trouvée par SONIA DOUAMBA qui trouve un âge moyen de 6,7 ± 2,9 ans pour la simple raison qu´elle avait travaillé sur les infections en pédiatrie [16]. Néanmoins son étude a également trouvé une forte prévalence d´infection chez les enfants de moins de 5 ans comme la nôtre. Manix ayant travaillé sur les complications digestives chez le drépanocytaire avait trouvé un âge proche au nôtre de 11,8 ans pour des extrêmes de 13 mois et 40 ans [9].

**En fonction du sexe:** nous avons trouvé une légère prédominance féminine avec une fréquence de 51,4% contre 48,6%. Cette légère prédominance est en accord avec plusieurs littératures traitant de la drépanocytose [16, 17] mais en contradiction avec certaines autres [18, 19] qui avaient trouvé une légère prédominance masculine; tout ceci prouve donc que la prédominance de l´un ou l´autre sexe est le fait du hasard et il n´y a donc pas d´explication scientifique qui justifierait la prédominance d´un sexe sur l´autre. D´ailleurs l´analyse des facteurs associés entre le sexe et les infections ostéo-articulaires montre que le sexe n´influence pas la survenue des infections ostéo-articulaires chez le drépanocytaire (p = 1,000).

**Concernant les antécédents**, la transfusion et la splénectomie ont été ceux qui nous ont intéressés dans notre étude. Sur un ensemble de 35 patients avec infection ostéo-articulaire, 3 seulement étaient splénectomisés et 32 ne l´étaient pas et 5 seulement avaient un antécédent de transfusion tandis que 30 n´avaient pas encore étaient transfusés. L´analyse des facteurs associés entre ces deux antécédents et la survenue des infections ostéo-articulaires chez les drépanocytaires ont montré qu´il n´y avait pas de différence statistiquement significative pour les deux antécédents (p = 0,090 et p = 1,000).

### Données cliniques

Concernant les motifs de consultation, la douleur a été le plus retrouvé avec 84,0% des cas ensuite l´impotence fonctionnelle avec 39% des cas. La douleur et la fièvre semblent être les motifs de consultation les plus rapportés dans la littérature [20]. Parfois la différence clinique n´est pas aisée entre l´infarctus osseux et l´infection osseuse car une douleur osseuse peut traduire à la fois une crise vaso-occlusive une ostéomyélite qui sont les deux complications les plus fréquentes obligeant une admission à l´hôpital des patients drépanocytaires [21, 22]. Berger E. [23] a rapporté dans son étude les facteurs prédictifs de l´ostéomyélite qui sont la fièvre et la douleur ainsi que la tuméfaction du membre atteint, le risque d´ostéomyélite est faible si plus d´un site de douleur est présent; la douleur osseuse et la tuméfaction affectant un seul site avec une fièvre prolongée doivent être considérées comme une ostéomyélite par le médecin et doit prescrire des investigations pour exclure le diagnostic d´ostéomyélite. Chambers rapporte que la douleur représente 79% des cas, la tuméfaction 71%, la fièvre 71% [14].

### Type d´infection

Nous avions sur nos 35 cas d´infections ostéo-articulaires une répartition de 24 cas d´ostéomyélite (68,57%) répartis en 18 cas d´ostéomyélite aiguë et 6 cas d´ostéomyélite chronique; 7 cas d´ostéite (20,00%) et 4 cas d´arthrite suppurée (11,43%). L´ostéomyélite est donc l´infection osseuse la plus retrouvée chez les drépanocytaires; ceci rejoint diverses littératures. Mary affirme que les infections ostéoarticulaires sont quasiment toutes des ostéomyélites (43%). L´ostéomyélite est donc l´infection osseuse la plus retrouvée chez les drépanocytaires; ceci rejoint diverses littératures. Mary affirme que les infections ostéoarticulaires sont quasiment toutes des ostéomyélites, les arthrites septiques vraies sont rares [20]. Une étude menée en Arabie Saoudite par Sadat Alim affirme que malgré l´amélioration des conditions socio-économiques de la communauté, l´ostéomyélite aiguë reste encore fréquente et sévère chez les enfants drépanocytaires [24] alors qu´une autre affirme que l´ostéomyélite diminue dans les pays développés; aux USA elle est estimée à 0,25% par Charles HJR *et al*. sur une population de 5900 drépanocytaires [25]. Et Marti-Carvajal affirme que les ostéomyélites (aiguës et chroniques) sont les principales complications infectieuses du drépanocytaire [26]. Les patients drépanocytaires seraient plus sensibles aux ostéomyélites et arthrites septiques que la population générale [27].

L´analyse des facteurs associés des types d´infection et l´âge a montré une forte prévalence de l´ostéomyélite chez les patients plus jeunes (17 cas sur les 24 chez les moins de 10 ans), l´arthrite était plus marquée chez les patients dont la tranche d´âge varie de 21 à 25 ans avec 2 cas sur 4. La différence est statistiquement significative (p = 0,0326). Nos résultats sont conformes à ceux de Nwadiarro [28] qui a trouvé 24 patients atteints d´ostéomyélite chronique sur un ensemble de 65 drépanocytaires soit 36,9% des cas; ce dernier affirme que l´incidence la plus élevée est retrouvée dans la première décennie de la vie (37,5% des cas). Ce qui se remarque dans plusieurs études ou la moyenne d´âge d´infection osseuse est de moins de 10 ans; Chambers (âge moyen: 8 ans), Sadat (âge moyen: 9,6 ans), Sonia Doumbia (âge moyen: 6,7 ans) [14, 16, 24].

### Localisation de l´infection

Toutes les localisations peuvent être touchées par l´ostéomyélite chez les enfants drépanocytaires mais les métaphyses fertiles des os longs sont les plus souvent concernés, les os longs développent souvent des pandiaphysites [29]. Les 4 cas d´arthrite suppurée ont été localisés principalement à la cheville avec 3 cas et le poignet 1 cas tandis que les atteintes osseuses sont réparties en 18 cas sur les os de la jambe dont 16 cas de tibia seul et 2 cas associant tibia et péroné, ensuite l´humérus avec 6 cas, les deux os de l´avant-bras radius et cubitus avec 5 cas, la main 1 cas et 1 cas d´infection du fémur. L´étude de Al-Salem a également trouvé une prédominance d´atteinte du tibia comme la nôtre mais chez lui le fémur et le l´humérus sont les plus touchés après le tibia [27]. L´étude de Sadat également trouve une prédominance d´atteinte du tibia avec 76 cas sur 201 [24] alors que celle d´Akakpo trouve une prédominance de l´humérus (28,7%), tibia (19,05%) et fémur (14,19%) [29]. Pour les arthrites; l´étude de Balogun avait trouvé par contre une prédominance de la hanche(40%), genoux(25%), coude(20%), épaule (10%) et cheville (10%) [30]. Nous pensons que la différence de ces résultats s´expliquerait par la différence importante de taille d´échantillon entre ces deux études.

### Les données de laboratoire

L´électrophorèse d´hémoglobine réalisée chez tous nos 35 patients a révélé une prédominance de la forme homozygote SS avec 88,6% des cas. L´analyse des facteurs associés du type de drépanocytose et de l´infection ostéo-articulaire montre qu´il existe une association statistiquement significative (p = 0,000). Chambers également trouve que tous les patients drépanocytaires ayant développé une ostéomyélite étaient des homozygote SS.

L´hémoculture avait été réalisée chez 17 patients et a isolé la *Salmonelle* chez 15 patients (soit 88,23%) et *Escherichia coli* chez 2 patients (21,77%) tandis que la pyoculture avait été réalisée chez 10 patients avec comme résultat 2 cultures stériles, 3 cas de staphylocoque doré, 2 cas de salmonelle et pneumocoque et 1 cas de *Klebsiella*; la *Salmonella* est donc le germe le plus retrouvé en cas d´ostéite ou ostéomyélite drépanocytaire dans notre milieu. Ce germe est également retrouvé chez plusieurs auteurs tels que Okuonghae [31] au Nigéria qui trouve une prédominance des salmonelle avec 25,9% des cas; Adeyokunnu [32] qui trouve 57 des 63 patients avec ostéomyélite à salmonelle était des drépanocytaires; Chambers qui trouve les salmonelles chez 8 malades drépanocytaires sur 10 (80%) atteints d´ostéomyélite, pour Juliana, la salmonelle est responsable de l´ostéomyélite dans plus de 50% et même à plus de 70% selon Onwubalili [33, 34]; mais signale qu´il n´y a pas de prédominance de germe dans les arthrites septiques [14]. Chez Diop, 21 cas d´infections à salmonelles (37%) et au niveau des os, 9 cas sur 11 d´infection osseuse à *Salmonella* [7]; les ostéites à *Salmonella* sont très fréquentes chez le drépanocytaire, le diagnostic est vite suspecté devant les signes d´appel [35]. Sadat également affirme que l´organisme le plus rencontré dans son étude était la *salmonelle spp*. chez 84 patients sur 201 soit 41,7% et dont 71 sur les 84 étaient des enfants de moins de 12 ans tandis que chez les adultes il s´agit d´autres germes [24]. A l´opposé, l´hémoculture et la culture des prélèvements du liquide ostéo-articulaire chez Sonia a isolé le *Streptococcus pneumoniae* (35,3%) et la salmonelle (33,3%) retrouvés à tout âge sans qu´il n´y ait une différence significative [16]. Contrairement à la multitude des résultats convergents sur la salmonelle, Nwadiaro trouve une forte fréquence de *Staphylocoque aureus* (58,8%) et ne trouve aucun cas d´ostéomyélite à salmonelle qui serait donc ainsi liée à l´endémicité du germe dans ces régions [28]; raison pour laquelle certains auteurs affirment que mise à part la forte sensibilité des salmonelles, les mauvaises conditions de vie de la population concernée permet d´expliquer une endémicité des salmonelles dans ces régions, le plus souvent des pays en voie de développement. Certaines études effectuées dans des pays développés n´ont pas trouvé de salmonelles [36-38]. Plusieurs hypothèses ont tenté d´expliquer la sensibilité des drépanocytaires à l´infection aux salmonelles, on pense au déficit chez le drépanocytaire en interféron gamma, substance qui inhiberait la multiplication cellulaire des salmonelles et à l´altération de la barrière intestinale provoquée par l´occlusion capillaire au cours des crises vaso-occlusives abdominales [24].

**Le bilan inflammatoire:** réalisé chez 21 patients et a révélé une hyperleucocytose au-delà de 15000 chez 15 patients (71,42%), une vitesse de sédimentation élevée chez tous les 21 patients (100%). L´hyperleucocytose est également retrouvée chez beaucoup d´auteurs, Sonia Douamba trouve 84,9% (16); mais il sied à signaler que l´hyperleucocytose est normale chez le drépanocytaire même en dehors de tout contexte infectieux. La VS élevée est également retrouvée chez la plupart d´auteurs, Chambers trouve 93% des patients avec une VS au-delà de la normale [14]. La radiographie: tous nos patients ont réalisé des clichés radiographiques. Celle-ci facilement accessible et à un prix abordable, nous permet ainsi de poser le diagnostic et de surveiller l´évolution des lésions. Les types d´images retrouvés sont les images d´ostéolyses (77,1% des cas), suiviesdes réactions périostées (15 cas soit 42,9%) et enfin les séquestres (6 cas soit 17,1%). L´atteinte osseuse classique de l´ostéomyélite aiguë, observée à la radiographie standard, est formée des lésions lytiques [12] justifiant notre fréquence élevée des lésions lytiques à la radiographie. La scintigraphie et l´imagerie par résonnance magnétique (IRM) sont mieux indiquées pour le diagnostic précoce des lésions osseuses qui prennent environ 2 semaines pour être visibles sur les radiographies; car le diagnostic précoce des infections ostéo-articulaires est la clé des résultats satisfaisants affirme Chambers [14]. Cependant l´indisponibilité de ces appareils dans notre milieu justifie sa non présence dans notre étude.

### Données thérapeutiques

Le traitement médical classique comprend une antibiothérapie, un antalgique auxquels tous nos patients sont soumis; l´unanimité quand à cet antibiotique n´est pas de mise à cause de quelques divergences sur les germes. Les céphalosporines de troisième génération ont été les antibiotiques les plus sensibles et donc les plus utilisés dans notre série, ceci rejoint les données de Sonia et Diagne chez qui également les céphalosporines de la 3^e^ génération ont été les plus utilisés [16, 18]. Il faut noter qu´il n´y a pas d´approche standardisée pour le traitement antibiotique qui varie sensiblement en fonction des régions [26] mais, chez les enfants, l´antibiothérapie devrait être dirigée contre les *salmonelles spp*. et chez les adultes couvrir également d´autres germes [24] alors que Al-Salem affirme que l´antibiotique utilisé doit couvrir les deux germes les plus retrouvés dans l´ostéomyélite et/ou l´arthrite septique chez le drépanocytaire qui sont la *salmonelle* et le *Staphylocoque aureus* [27]. Dans l´étude de Nwadiaro, le plus sensible était la gentamycine et les céphalosporines de la troisième génération [28].

**Le traitement orthopédique ou chirurgical:** le traitement chirurgical n´avait concerné que 6 patients qui avaient présenté des séquestres à la radiographie et a donc consisté en une séquestrectomie. Le drainage des arthrites suppurées chez 4 patients. La majorité de traitement était orthopédique avec la pose des plâtres selon les diverses localisations infectieuses. Notre traitement est en accord avec celui de Nwadiaro au cours duquel 15 cas d´ostéomyélite chroniques avec séquestres ont été traités par séquestrectomie et curetage mais également incision-drainage chez 4 autres patients [28]. Ensuite la surveillance est fonction des différentes radiographies réalisées tous les 1 mois. Le traitement agressif avec incision, forages osseux, drainage et antibiotique pendant 6 semaines est requis pour garder basse l´incidence des ostéomyélites chroniques chez les patients drépanocytaires [24]. Pour l´arthrite suppurée, le traitement a consisté en une évacuation du pus et rinçage articulaire au sérum physiologique par une arthrotomie avec drain laissé en place pendant 48 heures suivie d´une contention par attelle plâtrée et une antibiothérapie adaptée au résultat de la pyoculture plus antibiogramme. Notre traitement est en accord avec celui de Balogum qui avait réalisé pour les arthrites suppurées une immobilisation après une ponction drainage ou arthrotomie [30].

**Le traitement de fond de la drépanocytose:** 28 patients sur les 35 avaient été transfusés, tous avaient été mis sous acide folique. Cependant seulement 5 étaient sous hydroxyurée. Ce faible taux des patients sous hydroxyurée avait déjà été relevé par Manix *et al*. [9]. La couverture vaccinale ordinaire du PEV (Programme élargi de vaccination) était de 62,86%. Notre taux est faible par rapport à celui rapporté par d´autres études affirmant qu’en Afrique subsaharienne, le taux de couverture vaccinale des enfants drépanocytaires est satisfaisant, allant de 87% à 99% [39-41] et comme celle de Sonia au Burkina Faso trouve 81,2%. Malgré la gratuité de ces vaccins de PEV, nombreux enfants drépanocytaires de Lubumbashi n´ont pas accès probablement par manque de vulgarisation de l´importance de ce dit vaccin aux parents des enfants drépanocytaires. En ce qui concerne, les vaccins hors du PEV qui sont recommandés par l´OMS, ils ne sont pas pris en charge par notre Etat congolais et les parents doivent s´en procurer avec leur propre argent expliquant ainsi que seulement 6 patients sur 35 soit 17,14% en avait accès. Ce dernier taux est encore beaucoup plus faible que celui de Sonia au Burkina Faso qui trouve 36 patients sur 100 (36%) ayant bénéficié de cette vaccination spécifique du drépanocytaire [16] confirmant la conclusion de Manix et collaborateurs sur la pauvreté manifeste de la population congolaise [9] qui n´arrive pas à se procurer les traitements spécifiques pour cette maladie chronique.

## Conclusion

Les drépanocytaires sont susceptibles aux infections, celles osseuses sont parmi les plus redoutables au vu de leur fréquence et surtout de leur caractère invalidant en cas d´une prise en charge tardive ou inappropriée. La prévention doit passer par une politique de vaccination spécifique des drépanocytaires de Lubumbashi chez qui la couverture vaccinale est encore extrêmement faible.

### Etat des connaissances sur le sujet

- Les infections aux germes encapsulés sont fréquentes chez les drépanocytaires à cause de l´asplénie fonctionnelle;
- Les infarctus osseux et les ostéomyélites sont les atteintes osseuses les plus fréquentes chez les drépanocytaires;
- La salmonelle est le germe le plus retrouvé dans les infections osseuses chez les drépanocytaires.

### Contribution de notre étude à la connaissance

- Notre étude est la première réalisée dans notre région traitant des infections osseuses chez le drépanocytaire de Lubumbashi, elle servira de base de référence sur cet aspect jamais encore abordé chez nous;
- L´âge est corrélé de façon statistiquement significative à la survenue des infections ostéo-articulaires, le bas âge étant prédisposé aux ostéomyélites chez les drépanocytaires;
- La couverture vaccinale spécifique de la drépanocytose est très faible à Lubumbashi expliquant un taux élevé d´infection chez les drépanocytaires.
